# Data Mining Techniques Applied to Hydrogen Lactose Breath Test

**DOI:** 10.1371/journal.pone.0170385

**Published:** 2017-01-26

**Authors:** Cristina Rubio-Escudero, Justo Valverde-Fernández, Isabel Nepomuceno-Chamorro, Beatriz Pontes-Balanza, Yoedusvany Hernández-Mendoza, Alfonso Rodríguez-Herrera

**Affiliations:** 1 Department of Computer Languages and Systems, University of Sevilla, Sevilla, Spain; 2 Hispalense Pediatrics Institute, Sevilla, Spain; 3 Department of Networks, University Ciego de Ávila, Ciego de Ávila, Cuba; National Research Council of Italy, ITALY

## Abstract

In this work, we present the results of applying data mining techniques to hydrogen breath test data. Disposal of H2 gas is of utmost relevance to maintain efficient microbial fermentation processes.

**Objectives:**

Analyze a set of data of hydrogen breath tests by use of data mining tools. Identify new patterns of H_2_ production.

**Methods:**

Hydrogen breath tests data sets as well as k-means clustering as the data mining technique to a dataset of 2571 patients.

**Results:**

Six different patterns have been extracted upon analysis of the hydrogen breath test data. We have also shown the relevance of each of the samples taken throughout the test.

**Conclusions:**

Analysis of the hydrogen breath test data sets using data mining techniques has identified new patterns of hydrogen generation upon lactose absorption. We can see the potential of application of data mining techniques to clinical data sets. These results offer promising data for future research on the relations between gut microbiota produced hydrogen and its link to clinical symptoms.

## Introduction

Hydrogen breath test is a valid tool for the assessment of gut microbiome functional activity. The interest in evaluating this activity is currently increasing. The focus of our research is to extract new conclusions from these well-known data sources looking at them from a different perspective. This perspective is based on the use of tools well experienced in other research areas such as data mining.

Non-invasive assessment techniques of the gastrointestinal function are on the rise, although they have extensively been used in medical practice. Sampling of freely diffusible gases allows for a better understanding of physiology and pathology [1]. One of these sampling methods, hydrogen breath tests, measure the amount of H_2_ generated by gut resident bacteria after an orally given substrate, as part of this gas passes to blood and it is removed by breathing, where it can be easily measured. Hydrogen is generated during anaerobic metabolism, and the human body at rest does not have an anaerobic metabolism. Anaerobic bacteria preferentially metabolize sugar molecules. These molecules are broken down into short-chain fatty acids (SCFA), carbon dioxide (CO_2_) and hydrogen (H_2_) as result of fermentation. There is strong evidence that an increase in the exhaled hydrogen is in direct relation to anaerobic metabolic activity. The amount of gas produced is also connected with location of gut fermentation activity [2].

Lactose malabsorption is a common disorder associated to gastrointestinal disorders, with prevalence in the adult population reaching 80–90%. Lactose is a disaccharide present in milk, composed by glucose and galactose. Its absorption depends on the action of enzymes located in the small intestinal epithelium. In the case of malabsorption, sugars are fermented by colonic bacteria [3]. Malabsorption of lactose is, undoubtedly, the entity in which the use of hydrogen breath test has been more widely applied. These tests are considered to be safe and reliable, compared to other more invasive techniques, as jejunal biopsy. This is an indirect test with a good level of sensitivity (77.5%) and a an excellent level of specificity (97.6%) [4]. False negatives might appear, especially by the presence of intestinal flora not producing hydrogen due to the use of antibiotics. False positives are rare and occur in cases of bacterial overgrowth.

A deeper knowledge of H_2_ dynamics in gut is highly desirable. Disposal of H_2_ gas is fundamental for maintaining efficient microbial fermentation processes, but the microbial groups responsible for this function are present in low abundance. These hydrogenotrophic microbes include acetogens, methanogenic archaea (MA) and sulfate-reducing bacteria (SRB). Hydrogen sulfide (H_2_S) is a potent genotoxin and it is involved in the pathogenesis of chronic inflammatory disorders of the colon [5]. Therefore, identification of H_2_ generation patterns may contribute to the understanding of gut’s inflammation.

Dark fermentation is the fermentative conversion of organic substrate to biohydrogen. It is a complex process exhibited by diverse groups of bacteria, involving a series of biochemical reactions using three steps, which are similar to anaerobic conversion in a process independent of the presence of light.

The same interest is emerging in human health as H_2_ metabolism has been linked to processes as obesity, colon cancer or inflammatory bowel disease. This study is only the first step of a more complex research pipeline.

A better understanding of by-products of colonic fermentation is needed to get a deeper knowledge of interactions between human metabolome and resident microbiota metabolome. The greater part of this knowledge comes from in vitro assays. The real world of in vivo processes is far from being well known. In humans, parameters such as region of the colon, supply of endogenous substrates (proteins, urea and mucin) and bicarbonate secretion, can greatly interact with bacterial metabolic processes.

Patterns of H_2_ production may be linked to particular sets of species interacting together. Identification of H_2_ production patterns is an initial step towards a better understanding of gut as a dynamic system where equilibrium between gas generation and consumption contributes to health and disease.

In this work, we present the results of applying clinical data mining to the results of lactose hydrogen breath tests. Data mining is the automated analysis of data repositories in order to extract models representing knowledge [6]. In particular, clinical data mining is the application of data mining techniques to clinical data [7], with the goal of interpreting available clinical data. It allows for the creation of knowledge models and provides assistance to clinical decision making. In the last 10 years, there has been a growing interest in application of data mining techniques to clinical data. MEDLINE has seen a sharp increase by a 10-fold on number of papers having the term “data mining” in their title [7]. Although there are many publications related to application of hydrogen breath tests, no approaches have been found where data obtained from the tests are further analyzed using data mining techniques. Some previous successful applications of clinical data mining to volatile organic compounds from human exhaled air can be found in works published by Van Berkel *et al* [8], thus leading to the application of clinical data mining to hydrogen breath tests.

Application of data mining techniques to the results of lactose hydrogen breath tests has not been previously published in medical literature. We have been able to extract some conclusions which have been already presented in some conferences, and new hypothesis have been generated about the tests.

Hydrogen breath tests are good candidates to be analyzed with data mining techniques, since they provide a great amount of data, as they are a common practice and each test generates a vast amount of data. One of the main advantages of application of data mining techniques is the appearance of new hypothesis not previously considered [9]. Therefore, new knowledge may be extracted from the data. New H_2_ generation patterns become unveiled and this could be connected, in theory, with inflammation mechanism and clinical symptoms.

The rest of the article is structured as follows. In Section Material and Methods we describe both hydrogen breath tests and data mining techniques, as well as the dataset analyzed. In Section Results we show our findings after application of data mining techniques. In Section Discussion, we extract conclusions about the results, and we talk about future lines of work.

## Material and Methods

Data sets from 2751 lactose hydrogen breath tests were included (see data in S1 Data Hydrogen breath test data). Collection time ranges over 4 years, from June 2009 to June 2013. Both genders subjects are between 1 and 14 years old. The information acquired for each patient was: gender, date of the test, age of the patient at the time of the test, weight, height, private assurance company name and zip code.

The research was conducted conforming to the principles outlined in the Declaration of Helsinki. Written informed consent was obtained from parents/guardian of each children enrolled. Clearance for this human subject research project has been given by *Fundación IHP Institutional Review* with level of review “Exempt, Non-Significant Risk(NSR)”.

### Breath Test Methods

Patients were fasting overnight, at least during 6 hours (depending on their age). Children who received antibiotics during the 2 weeks prior to testing were excluded, as well as patients with intra-abdominal surgical proceedings conducted 4 weeks before the test. Patients who had used laxatives in the 3 days prior to the test were also excluded. Patients were advised to avoid slowly absorbed carbohydrates (like bread or potato) and fiber the night before the test.

Breath samples were taken by facial mask for children with blowing difficulties or by disposable card tubes. Breath sample*s* were analyzed using a portable device (Gastrolyzer®, Bedfont Scientific *L*td). Samples were taken at baseline (0 minutes) and minutes 30, 60, 90, 120, and 180 after ingestion of a 50g/250 mL ready-to-use lactose solution (Lactonaranja®, Bioanalitica SL, Spain). Gastrointestinal discomfort symptoms were checked along with each of the samples taken. Patients were kept under direct supervision for the whole process to avoid excessive exercise that could interfere with the readings.

The Rome Consensus Conference evaluated the methodology and indications of lactose breath tests in gastrointestinal disorders and suggested a cut-off increase of hydrogen of 20 parts per million (ppm) above the baseline level to be considered as positive [2]. We refer to malabsorption when such increase occurs.

### Data Visualization

This section provides some graphical information about the dataset analyzed. In particular, two different analysis has been carried out.

**Fig 1:**
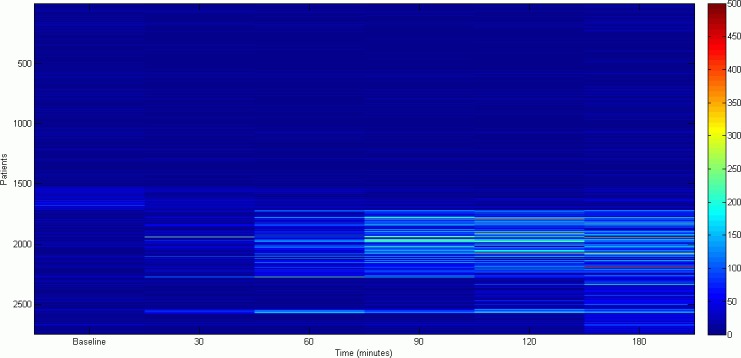
Data visualization by means of a heat map.

First, a simple visualization of data heat map can be found in Fig 1. We can see for some of the patients a high variability in values, ranging from 0 to almost 500 ppm. Additionally, it can be seen that higher variations typically occur at the sample at 180 minutes.

**Fig 2:**
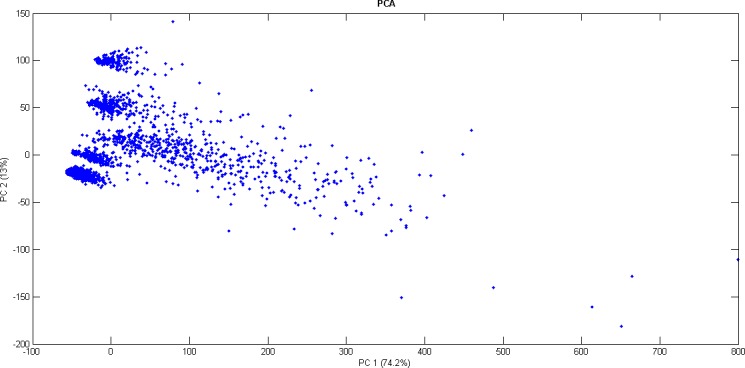
Data visualization of the first two principal components.

Additionally, a principal component analysis (PCA) [10] has been carried out. This study aims at a priori determination of the number of possible existing partitions. Fig 2 illustrates data distribution into the first two components, which contain 87.2% of data variability (74.2% and 13%, respectively). A third component containing 5.8% of variability was also discovered.

This figure suggests that there are five partitions clearly differentiated and the existence of a sixth partition which is spread in the space. This sixth partition will present higher intra-cluster distance. Furthermore, such shapes also suggest that the extracted clusters exhibit spherical distribution. Therefore, k-means is proposed as the clustering algorithm using the Euclidean distance.

### Data Mining Methods

Clustering analysis or clustering is an exploratory data mining task used in many fields such as machine learning, pattern recognition or information retrieval [11]. Clustering is the process of grouping a set of data objects (or examples) into subsets. Each subset is named cluster and the objects in a cluster are similar to one another, yet dissimilar to objects in other clusters. The set of clusters resulting from a cluster analysis is named also as clustering. The process of grouping or partitioning is performed by clustering algorithms in an automatic way. Clustering is one of the main tasks in data mining and it can lead to the discovery of previously unknown groups within the data [12].

There are many clustering algorithms in the literature [13]. In this work, we use the k-means algorithm [14], which is a partitioning method. The justification for this selection can be found in Section Data Visualization. Partitioning methods divide the objects into k partitions or groups with the restriction of k< = n, where k is the number of clusters to discover, and n the number of objects in the dataset D.

Most partitioning methods are distance-based methods. Given k, the method creates an initial partitioning and it uses an iterative process that tries to improve the partitioning by moving objects from one group to another based on a distance measure. The criterion for a good partitioning is that objects in the same group are similar or close given a distance measure, whereas objects in different clusters are very different or far apart.

K-means is the most well-known and commonly used partitioning method [15]. Given a dataset D with n objects and a k value, the partitioning method distributes the objects from D in k clusters: C_1_, C_2_…, C_k_, where C_i_ and C_j_ are included in D and C_i_ and Cj have an empty intersection. K-means can be used with different distance measures, being the Euclidean distance [16] the most commonly used, and therefore the one used in this work.

K-means is a centroid-based technique and this technique uses the centroid of a cluster, Ci, to represent the cluster. Basically, the centroid of a cluster is its center point and can be defined in several ways, for instance using the means or medoid of the objects assigned to the cluster. In the case of k-means algorithm, the means of the objects are used as a measure to define the center point. The difference between two objects p and q in cluster C_i_ is measured by the Euclidean distance between these two points with the centroid.

K-means algorithm proceeds as follows: the algorithm randomly chooses k objects in D and these objects initially represent the centroids of the k clusters. For each of the remaining objects, the Euclidean distance is calculated to assign each object to the most similar cluster. Then, the cluster centroid is recalculated and the objects are reassigned only if the sum of distances is reduced. The clusters centers are recalculated after each reassignment. The algorithm stops when moving objects from one cluster to another does not reduce the sum of distances, i.e., each object is grouped with its most similar or closest objects.

A hot topic in clustering processes is the selection of the optimal number of clusters. Some methods such as Expectation-Maximization provide the user with the best number of clusters. Unfortunately, this is not the most usual situation and, in many cases, the user must discover this value. Several methods have been proposed so far, some of them focusing on inter-cluster distances, some others in intra-cluster distances or, in other cases, combinations of both distances and other considerations. In [17], a method combining several indices was proposed. In particular, the authors used the well-known measures Silhouette [18], Davies-Bouldin [19] and Dunn index [20] to create a system based on a majority of votes to determine the most suitable number of clusters. We are following this strategy in this work.

Additionally, k-means has another important issue to be addressed: its sensitivity to the starting partition. To prevent local minima, the k-means++ algorithm [21] for cluster center initialization has been used. This algorithm improves the running time of the k-means algorithm, and the quality of the final solution due to the heuristic it uses to find centroid seeds.

Finally, please note that all experimentation has been carried out in Matlab 2015, in a i7 QuadCore 3.30 GHz processor, using the routines provided by Matlab and taking less than 3 minutes to perform the whole experimentation.

## Results

The data set was made up of 2751 hydrogen tests fulfilling inclusion criteria. From them, 181 were excluded because of missing data. Following the Rome Consensus, we considered as Lactose Malabsorption (LM) samples from patients showing an increase of hydrogen levels of 20 parts per million (ppm) above the baseline. The number of patients diagnosed of lactose malabsorption (LM) following these criteria was 839, 32.64% of all patients. In our study, we have not discriminated non-hydrogen producing patients to avoid loses due to selection bias.

Patients with an initial H_2_ basal value greater that 20 ppm were considered, although these patients are sometimes discarded for the test. There is controversy over the interpretation of a baseline above 10-20ppm. Some authors consider the test as invalid due to slowed gastric emptying and oral flora effect, discontinuing the test [22]. Other authors consider this finding as suggestive of bacterial overgrowth and continue taking samples. There were 93 patients with initial H_2_ basal value greater than 20 ppm diagnosed of lactose malabsorption. They represented 3.62% out of a total set of 2571 patients analyzed and 11.08% out of 839 patients diagnosed as intolerant.

To highlight the relevance of the different samples taken throughout each of the tests, we calculated how many of the patients would have been diagnosed of malabsorption if some of the samples had been ignored. Our finding was that excluding samples at 90 minutes, 19 patients would have not been diagnosed of malabsorption, representing 2.26% of the 839 patients diagnosed of LM. If we do not take into account samples at 120 minutes, 26 patients would have not been diagnosed of malabsorption, representing 3.09% of the 839 patients diagnosed with malabsorption. Finally, for sample at 180 minutes, 166 patients would have not been diagnosed of malabsorption, representing 19.78% of the 839 patients diagnosed with malabsorption.

Regarding data mining techniques, we applied k-means clustering to the data set in order to identify patterns of behavior. Not previous hypothesis were considered. The clustering was applied to the H_2_ values, removing the the one at minute 150. K-means needs as input parameter the number of groups or clusters k in which dividing the dataset. The k-means algorithm was executed with k = 2, 3, 4, 5, 6, 7 and 8. The initial value k = 2 was chosen since classically, hydrogen lactose breath tests, provide results based on 2 types of curves [22]. The last evaluated value k = 8 has been chosen based on expert knowledge.

**Fig 3:**
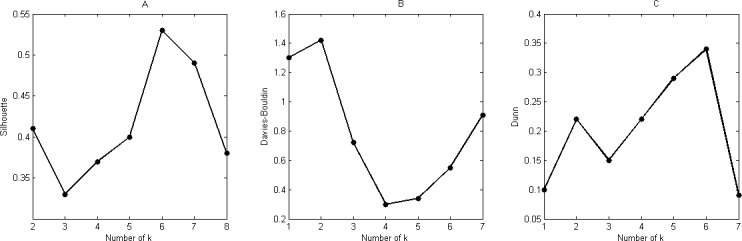
Results for Silhouette (4A), Davies-Bouldin (4B) and Dunn (4C) for k = 2 to k = 8.

According to the methodology described in [17], values for Silhouette, Davies-Bouldin and Dunn Index are represented in Fig 3, for k = 2 to k = 8, as aforementioned (calculated with Matlab’s package *‘CVAP*: *Cluster Validity Analysis Platform’*). Since all indices represent results at different scales, we have preferred to show them in their original scale.

Table 1 reports the best and second best results for all the three methods. Silhouette and Dunn aims at maximum values, whereas Davies-Bouldin aims at low values. If only the best values were considered (k = 6, k = 5, and k = 7, respectively) no consensus would be reached. However, when considering the second best values (k = 7, k = 6, and k = 6, respectively), k = 6 is finally elected with 3 votes.

**Table 1:** Selection of the most suitable number of clusters.

Selection of k	Silhouette	Davies-Bouldin	Dunn
Best	k = 6	k = 5	k = 7
Second best	k = 7	k = 6	k = 6

**Fig 4:**
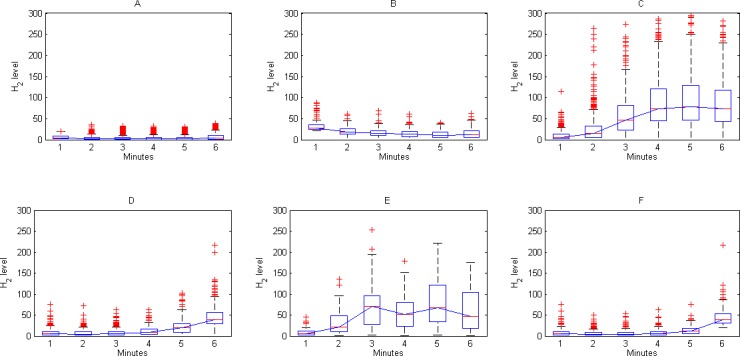
Graphical representation of the boxplots and median values of the groups obtained.

Therefore, the most suitable partitions are obtained when k = 6, identifying a set of 6 different typologies of data curves (see Fig 4):

- A. Flat pattern, non-ascending, linked to baseline lower than 20 ppm (1602 patients, 58.36%).
- B. Flat pattern, non-ascending, linked to baseline over 20 ppm (223 patients, 8.12%).
- C. Curved pattern, ascendant before sample at 90 minutes (121 patients, 4.41%).
- D. Curved pattern, ascendant after sample at 90 minutes (518 patients, 18.87%).
- E. Curved pattern, doubly ascendant, before and after sample at 90 minutes (121 patients, 4.41%).
- F. Curved pattern, ascendant only at sample at 180 minutes (166 patients, 6.05%).

Furthermore, a one-way ANOVA has been applied in order to confirm the statistical presence or absence of average differences among the time points within the clusters (function *‘anova1’* in Matlab). Table 2 summarizes ANOVA results, showing the between-groups variation (Columns) and within-groups variation (Error). SS is the sum of squares, and df represents the degrees of freedom. MS is the mean squared error, which is SS/df for each source of variation. The F-statistic is the ratio of the mean squared errors. The *p*-value is the probability that the test statistic can take a value greater than or equal to the value of the test statistic, and the value to reject the null hypothesis is *p*-value < 0,05. The small *p*-value of 2.67e-159 indicates that the null hypothesis of equal averages for the time points within the clusters can be rejected.

**Table 2:** Summary of ANOVA results among the time points within the clusters.

Source	SS	Df	MS	F	Prob > F
Columns	1.05e+06	5	210850.9	152.92	2.67e-159
Error	2.27e+07	16464	1378.8	--	--
Total	2.38e+08	16469	--	--	--

In order to provide a more comprehensive analysis of the reported results, metadata has been studied as well. These studies are of particular interest and provide very useful information [23]. Please note, that all patients were characterized by gender, age, weight and height. However, too many missing values were encountered in both weight and height to be considered in this study and they were not included in this analysis. Table 3 summarizes age and gender distribution into clusters.

**Table 3:** Patient distribution into average age and gender, according to discovered clusters.

Feature	Cluster A	Cluster B	Cluster C	Cluster D	Cluster E	Cluster F
Age average (years)	4.99	5.05	7.53	5.24	5.95	5.09
Males (%)	50.23	70.59	54.53	49.79	52.63	52.08
Females (%)	49.77	29.41	45.47	50.21	47.37	47.92

Regarding the age in cluster distribution, we see that the average is higher for cluster C. One-way ANOVA has been again applied (see Table 4) to confirm the different age distribution within clusters, obtaining a *p*-value of 0. This value indicates that the null hypothesis of equal averages for the age within the clusters can be rejected. However, the ANOVA does not tell you where the difference lies. We then applied Student’s t-Test to test each pair of averages, finding the different age average distribution for cluster C. The resulting tables are not included for legibility reasons.

**Table 4:** Summary of ANOVA results among the age distribution within the clusters.

Source	SS	Df	MS	F	Prob > F
Columns	26453.8	5	5290.75	412.18	0
Error	89171.2	9138	9.76	--	--
Total	1156725	9143	--	--	--

These findings may indicate that it is more common to present curve type C at an older age. This kind of curve suggests bacterial overgrowth or accelerated intestinal transit, which could be justified related to a more mature system.

The gender distribution is also different for cluster B. There is a considerably higher percentage of males than females in this group. This could be related to males being more likely to perform an incorrectly test preparation or to present a slow transit or a bacterial overgrowth. The last result might also refer to a difference in dynamics in pulmonary ventilation rather than a real difference in intestinal fermentation, as some studies suggest that pulmonary parameters differ according to gender in children [24].

## Discussion

Data mining techniques are increasing their presence in practical clinic, complementing classical statistics analysis. Data mining is particularly useful when data volume increases [25]. Among all test available involving exhaled hydrogen, the lactose ones are the most widely used and with more scientific evidence, and there is no doubt about its clinical utility [26]. Lactose hydrogen breath is a well stablished test that, due to the results yielded (numerical values throughout time), is a good candidate for data mining analysis. As far as we know, this work is the first one to apply data mining to lactose hydrogen breath test results.

After analyzing the 2571 tests contained in the data set using data mining techniques, we have identified six different patterns (see Fig 4). Among them, two are the types of curves classically identified [27]: a flat pattern without rise (See Fig 4A), yielding a negative test and a curved pattern with a rise of at least 20 ppm from the baseline value beyond sample at minute 60 (see Fig 4D).

What is new is the identification of other four patterns of behavior. Although these curves are referenced in literature [2], they are not considered classically as independent patterns of presentation for hydrogen breath tests results. Interpretation of curves with a high level of H_2_ at the baseline sample and no further rise in the other samples (see Fig 4B) is controversial. Upon verification that no rule violations in pre-procedure conditions are responsible for this baseline high level, some authors consider the test invalid and decide to repeat it after some weeks. Others consider this finding suggestive of bacterial overgrowth and slow transit and complete the test in the standard way [28]. In our case, the high H_2_ baseline level has not interfered in diagnosis of LM. So we consider that the test should not be discontinued by this finding, as it maintains its diagnostic value regardless of the baseline value.

The pattern rising before the sample at 90 minutes (see Fig 4C) is not considered as diagnostic of malabsorption. It has been associated to rapid intestinal transit or the existence of bacterial overgrowth (SIBO). As in the previous case, the bacterial overgrowth can be confirmed by alternative tests [29]. These findings are not enough to get a well establish diagnostics of SIBO, because lactose test is not appropriate for this case.

The meaning of the double-hump shape is uncertain (see Fig 4E). It seems to be a combination between the lactose intolerance curve (Fig 4D) and the curve described above (Fig 4C). These patients are usually diagnosed of intolerance and it is necessary to confirm the presence or not of overgrowth with a specific test.

Finally, we would like to focus on the curve with a rise only at sample at 180 minutes (see Fig 4F). Finding this curve confirms the relevance of extending the test to 180 minutes [2] against other publications that limit the test to 120 minutes [27]. Some sampling centers follow this shortened pattern for cost-saving reasons. We consider that this practice greatly increases the rate of false negatives, as in our study more than 10% of the patients with LM exhibited this pattern.

It would have been of great interest to correlate the test results with the clinical symptoms showed by patients during the test, or after a trial on lactose-free diet. Due to design and funding limitations, it was unfeasible. Prospective studies in this field, taking into account the findings observed, would be of great interest. Now, with clearly established patterns, it will be possible to design a research project to correlate signs and symptoms.

We would like to emphasize the fact that, as far as we know, this work is the first one to apply data mining techniques to hydrogen breath test results. It has provided six previously unidentified patterns of gas production. As future research, we propose linking these six patterns to different sets of symptoms or metabolic activity of gut flora. This may be considered as a step into basic research that needs to move further to applied research.

The colonic microbiota has been characterized from stool samples studies. Analysis of metabolite production patterns as H_2_ may be an easy to use tool to get deeper knowledge of functional status of flora. H_2_ tests are widely available and un-expensive.

Hydrogen metabolites, as H_2_S, have been proposed as *gasotransmitter*. These are gaseous signaling molecules that are either synthesized endogenously in the organism, tissue or cell or are received by the organism, tissue or cell from outside. Intestinal microbiota is one of its potential sources. They work transmitting chemical signals which induce certain physiological or biochemical changes in tissues or cells. H_2_S has been found to have dichotomous effects, acting as and stimulatory as well as an inhibitory molecule. It is involved on several gastrointestinal processes such as inflammation, contractile responses, nociception, cancer and apoptosis. Bacterial fermentation of complex carbohydrates in the colon releases large amounts of hydrogen, which is consumed by hydrogenotrophs that include methanogens acetogens and sulfate reducing bacteria (SRB). Analysis if flatus composition of human subjects revealed H_2_S concentrations in the range 0.2 to 30 ppm. A high level of H_2_S induces DNA damage, inhibits cytochrome C oxidase, and inhibits butyrate oxidation [30].

The equilibrium between H_2_ generation and its use by hydrogenotrophic microbes may be assessed through analysis of breath test curves shape. Diversity and ecology of mucosa-associated microbes may be deducted from this pattern and potentially maybe related to mechanism of health and disease. This new approach is closer to the practical clinical field, as H_2_ measuring equipment is much widely available.

Molecular hydrogen might affect a number of enteric bacterial infections. This is indicated by genetic evidence for hydrogen-consuming hydrogenases, in vitro data demonstrating roles of hydrogenases in energy conservation, metabolite uptake, and acid resistance by various enteropathogens, including *E*. *coli*, *Shigella spp*., *Yersinia spp*., and *Campylobacter spp*.

The intestinal microbiota features include metabolic interactions involving the breakdown and reuse of host and diet-derived nutrients. The competition for these resources can limit pathogen growth. Thus, H_2_ has been proposed to act as an Achilles’ heel of microbiota metabolism that can be modulated by pathogens and might offer opportunities to prevent infection [31]. So the need to know its cycle of generation and consumption arises, and this study is a contribution to it.

Breath hydrogen testing has been incorporated into hundreds of published research studies. But surprisingly, the approach has been quite conservative, with a limited interpretation of data and a lack of correlation between H_2_ generation and dynamics of gut microbiota metabolome actions. New research lines can be designed after description of H_2_ production patterns, focusing now on the biochemical reactions that generate H_2_ by different bacterial species and how it may be modulated.

These results open the field for future research on the relationship of these patterns with clinical symptoms, intestinal microbiota, motility and other aspects of interest. Data mining yields new insights from easily accessible clinical data and unveil conclusions not evident before.

## Supporting Information

S1 Data Hydrogen breath test data. This is the data used for the research presented in this manuscript.
(XLSX)
